# Hearing loss grades and the *International classification of functioning, disability and health*

**DOI:** 10.2471/BLT.19.230367

**Published:** 2019-09-03

**Authors:** Bolajoko O Olusanya, Adrian C Davis, Howard J Hoffman

**Affiliations:** a Centre for Healthy Start Initiative, 286A Corporation Drive, Dolphin Estate, Ikoyi, Lagos, Nigeria.; b The Ear Institute, University College London, London, England.; c Division of Scientific Programs, National Institute on Deafness and Other Communication Disorders, Bethesda, United States of America.

Hearing impairment negatively affects well-being and is a major contributor to years lived with disability.^1^^,^^2^ The World Health Organization (WHO) estimates that 466 million people were living with disabling hearing impairment in 2018 and this estimate is projected to rise to 630 million by 2030 and to over 900 million by 2050.^3^ However, these projections are based on a hearing impairment classification that does not fully reflect the provisions of the *International classification of functioning, disability and health* for assessing all forms of functional impairments.^4^ Here we make the case for a review of the concept of disabling hearing loss adopted by WHO after the recommendation of the Global Burden of Disease (GBD) Expert Group on Hearing Impairment in 2008.

The need for an independent classification system for all impairments and disabilities as a complement to the well-established *International statistical classification of diseases and related health problems*, was first suggested in 1976 by the World Health Assembly. As a result, in 1980 WHO developed the *International classification of impairments, disabilities and handicaps*.^5^ One of the key features of this system was the use of qualifiers such as mild, moderate, severe and profound to distinguish various levels of observed or measured deviations outside of the range considered for normal functioning for any health condition. This categorization has been reinforced in the subsequent revisions to the system, such as the *International Classification of Functioning, Disability and Health*, and accompanied with descriptions of typical problems encountered in daily activities at various levels of severity.^4^ The classification, notably, does not use the term disabling, as it recognizes the needs of all persons with functional impairments for appropriate intervention.

WHO’s first classification for hearing impairment dates to 1986 and is based on the recommendations of an expert group established by WHO specifically for this purpose; the classification has been modified several times since then. The current one is shown in Table 1; it is based on the version published in 1991, which remarked that persons with average pure-tone audiometry of 15–20 decibels (dB) hearing level may experience hearing problems, and those with unilateral hearing losses may experience hearing problems even if the better ear was normal.^6^ However, currently only adults (≥ 15 years) with a permanent unaided hearing impairment above 40 dB hearing level in the better ear and children (from birth to 14 years of age) with > 30 dB hearing level are regarded as having a disabling hearing impairment.^3^ This classification raised three major concerns. First, the prescribed threshold for normal hearing of 25 dB hearing level is not in agreement with several reports in the literature on the functional experience of persons with slight or mild hearing impairment (< 25 dB hearing level). Second, there is no scientific or rational basis for the uneven steps between the various grades of severity. Third, and more crucially, the definition of disabling hearing impairment excludes all persons with unilateral hearing impairment of any severity and those with mild bilateral hearing impairment, which is not consistent with the *International classification of functioning, disability and health*.

**Table 1:** WHO’s Grades of hearing impairment

Grade of impairment	Corresponding audiometric ISO value^a,b^	Performance	Recommendations	Comments added to the previous classification
0: no impairment	25 dB or better	No or very slight hearing problems. Able to hear whispers	None	20 dB also recommended. People with 15 – 20 dB levels may experience hearing problems. People with unilateral hearing losses may experience hearing problems even if better ear normal
1: slight impairment	26–40 dB	Able to hear and repeat words spoken in normal voice at 1 m	Counselling. Hearing aids may be needed	Some difficulty in hearing but can usually hear normal level of conversation
2: moderate impairment	41–60 dB	Able to hear and repeat words using raised voice at 1 m	Hearing aids usually recommended	None
3: severe impairment	61–80 dB	Able to hear some words when shouted into better ear	Hearing aids needed. If no hearing aids available, lip-reading should be taught	Discrepancies between pure-tone thresholds and speech discrimination score should be noted
4: profound impairment including deafness	81 dB or greater	Unable to hear and understand even a shouted voice	Hearing aids may help in understanding words. Additional rehabilitation needed. Lip-reading and sometimes signing essential	Spoken speech distorted, the degree depending on the age at which hearing was lost

dB: decibel; Hz: Hertz; ISO: International Organization for Standardization; m: meter; WHO: World Health Organization.

^a^ In the better ear.

^b^ Average of 500, 1000, 2000 and 4000 Hz.

Notes: Disabling hearing loss refers to hearing loss greater than 40 dB in the better hearing ear in adults (Grades 2, 3 and 4) and greater than 30 dB in the better hearing ear in children.

Source: WHO.^6^

In 2008, the GBD Expert Group on Hearing Loss addressed these concerns by reviewing the WHO classification for hearing impairment and other data inputs for the GBD study 2010.^7^ This assignment resulted in a proposal for a revised classification (Table 2). The limit for normal hearing was reduced from 25 to 20 dB hearing level, a separate category for unilateral hearing impairment was introduced and the six categories for bilateral hearing impairment were differentiated consistently by 15-dB steps. The decision to reduce the limit for normal hearing was informed by the extensive clinical experience of this expert group as well as available evidence in the literature at the time. While threshold of normal hearing set at 15 dB could be found in the literature, often resulting in an additional category of slight impairment (16–25 dB hearing level),^8^ the expert group opted not to deviate from the *International classification of functioning, disability and health* lowest generic qualifier of mild impairment, but reduced the lower limit from 25 to 20 dB hearing level. This decision was reinforced by the fact that persons with hearing sensitivity < 20 dB rarely benefit from or require amplification devices, and was in agreement with the remarks by the WHO expert group in 1991.^6^ For example, one survey showed that only 2.7% of a total of 556 026 hearing aids dispensed over an undisclosed period were for persons with < 20 dB hearing loss.^8^ Perhaps, most notable was the predominant use of this threshold in sweep test audiometry, especially in school-aged children.^9^

**Table 2:** Grades of hearing impairment as recommended by the Global Burden of Disease Expert Group on Hearing Loss

Category	Pure-tone audiometry^a,b^	Hearing experience in a quiet environment	Hearing experience in a noisy environment
Normal hearing	−10.0 to 4.9 dB hearing level	Excellent hearing	Good hearing
	5.0 to 19.9 dB hearing level	Good hearing	Rarely have difficulty in following/taking part in a conversation
Mild hearing loss	20.0 to 34.9 dB hearing level	Does not have problems hearing what is said	May have real difficulty following/taking part in a conversation
Moderate hearing loss	35.0 to 49.9 dB hearing level	May have difficulty hearing a normal voice	Has difficulty hearing and taking part in conversation
Moderately severe hearing loss	50.0 to 64.9 dB hearing level	Can hear loud speech	Has great difficulty hearing and taking part in conversation
Severe hearing loss	65.0 to 79.9 dB hearing level	Can hear loud speech directly in one’s ear	Has very great difficulty hearing and taking part in conversation
Profound hearing loss	80.0 to 94.9 dB hearing level	Has great difficult hearing	Cannot hear any speech
Complete or total hearing loss	95.0 dB hearing level or greater	Profoundly deaf, hears no speech or loud sounds	Cannot hear any speech or sound
Unilateral	< 20.0 dB hearing level in the better ear, 35.0 dB hearing level or greater in the worse ear	Does not have problems unless sound is near poorer hearing ear	May have real difficulty following/taking part in a conversation

dB: decibel; Hz:Hertz

^a^ In the better ear.

^b^ Average of 500, 1000, 2000 and 4000 Hz.

Source: Global Burden of Disease Expert Group on Hearing Loss.^7^

A separate category for unilateral hearing impairment was introduced in line with the extensive literature on the functional, educational, psychological or social impact of this type of hearing impairment in all age groups.^10^ This category also reflected the *International classification of functioning, disability and health* specific provisions for hearing problems associated with localization and lateralization, particularly in difficult listening situations. The GBD expert group proposed six categories for bilateral hearing impairment, differentiated consistently by 15-dB steps, since scientific or clinical evidence was lacking to support the variable steps between the grades in the WHO classifications. The choice of 15-dB was intended to reflect the minimum shift in pure-tone audiometry thresholds that is typically considered as clinically and functionally significant, especially in occupational noise surveillance.^11^

The concept of disabling hearing impairment also needed to be appropriately expanded to include both unilateral and bilateral hearing impairment outside the range for normal hearing. This was to ensure that the persons with such impairment were not discriminated against in accessing appropriate interventions. Calibrating the degree of difficulty in percentage terms across the various categories as suggested in the *International classification of functioning, disability and health* is difficult. However, by definition, a condition is said to be disabling if it causes someone to have an illness, injury or condition that makes it difficult to do the things that other people do. As a result of the expanded concept of disabling hearing impairment, it was no longer critical to have a separate classification for children (Table 1).

However, the term disabling is neither recommended by *International classification of functioning, disability and health* nor routinely applied for other impairments. Therefore, discontinuing such description might be more appropriate from a practical, human rights and equity perspective. Conceiving an impairment or disability that is not disabling in all contextual factors is challenging. However, discontinuing the use of the term should not prevent a public health policy that prioritizes those for whom hearing devices (hearing aids and cochlear implants) would provide more benefit, especially in low-resource settings. The mere recognition of those with mild hearing impairment could be advantageous, especially for school children who may benefit from simple but effective intervention such as preferential seating in the classroom. Such recognition would also help parents and carers to have better understanding of the communication challenges children with mild hearing impairment face in their daily life.

Classifications based solely on pure-tone audiometry have limitations.^8^ For instance, while measurement of a behavioural pure-tone audiometry tests the entire auditory pathway, it does not localize to any one segment along the auditory pathway. Hence, this measurement does not provide specific information on the status of the central auditory nervous system and, therefore, may offer only limited insight into auditory function in real-world settings. Therefore, persons with normal pure-tone audiometry thresholds can report having significant difficulties in hearing. Concerns also exist regarding the various qualifiers attached to pure-tone audiometry thresholds such as normal hearing or mild hearing impairment, as these qualifiers do not always preclude some degree of functional impairments.^8^ As a result, the proposed pure-tone audiometry categories should be complemented with descriptions of functional performance that are not offensive or derogatory in any given cultural context and that reflect activity limitation or participation restriction, particularly in quiet and noisy environments. These categories should also include possible indications for rehabilitation with assistive devices. Moreover, the *International classification of functioning, disability and health* allows that, wherever possible, the person whose level of functioning is being classified (or the person’s advocate) should have the opportunity to participate and to challenge or confirm the appropriateness of the category being used, and the assessment assigned.

The proposed classification was first reported in the 2010 GBD study to generate prevalence estimates and disability weights for hearing impairment, and has been adopted in all subsequent GBD publications.^2^ A recent study specifically investigated the extent to which the revised pure-tone audiometry classification correlated with communication deficits based on functional measures of speech communication in the adult population.^12^ The analysis showed good validity for the proposed classification system, based on the evidence from relatively large population and clinical studies. The analysis also established significant changes in functional communication as the classification progresses from slight/mild through severe grades.

We recommend that WHO considers adopting this revised classification to ensure that future WHO estimates of hearing impairment are aligned with those published periodically in the GBD study and other epidemiological data. Doing so would also ensure that no persons with functional hearing impairment, regardless of the severity, are unduly placed at a disadvantage compared to those with other impairments within the framework of the *International classification of functioning, disability and health*, especially under the disability-inclusive global agenda of the sustainable development goals.
